# Prevention of cerebral thromboembolism by oral anticoagulation with dabigatran after pulmonary vein isolation for atrial fibrillation: the ODIn-AF trial

**DOI:** 10.1007/s00392-023-02319-9

**Published:** 2023-11-03

**Authors:** Jan Wilko Schrickel, Thomas Beiert, Markus Linhart, Julian A. Luetkens, Jennifer Schmitz, Matthias Schmid, Gerhard Hindricks, Thomas Arentz, Christoph Stellbrink, Thomas Deneke, Harilaos Bogossian, Armin Sause, Daniel Steven, Bernd-Dieter Gonska, Boris Rudic, Thorsten Lewalter, Markus Zabel, Tobias Geisler, Burghard Schumacher, Werner Jung, Thomas Kleemann, Armin Luik, Christian Veltmann, Martin Coenen, Georg Nickenig

**Affiliations:** 1 Department of Cardiology-Rhythmology, Marienhospital Siegen, Germany; 2 Department of Medicine-Cardiology, University Hospital Bonn, Venusberg-Campus 1, 53127 Bonn, Germany; 3 Secció d’Arrítmies, Cardiologia Hospital Universitario *de Girona Doctor Josep Trueta, Girona, Spain; 4 Department of Diagnostic and Interventional Radiology, University Hospital Bonn, Bonn, Germany; 5 Institute for Medical Biometry, Informatics and Epidemiology, University Hospital Bonn, Bonn, Germany; 6 Department of Rhythmology, DHZC, University Hospital Charité, Berlin, Germany; 7 Heart Center Freiburg, University Bad Krozingen, Bad Krozingen, Germany; 8 Department of Cardiology and Intensive Care Medicine, University Hospital OWL Campus, Bielefeld, Germany; 9 Clinic for Cardiology II, Heart Center Bad Neustadt-Saale Bad, Neustadt, Germany; 10 Medical Clinic III Hospital Lüdenscheid, Lüdenscheid, Germany; 11 University of Witten-Herdecke, Witten, Germany; 12 Department of Cardiology, Helios Hospital Wuppertal, Wuppertal, Germany; 13 Department of Electrophysiology, University Hospital Cologne, Cologne, Germany; 14 Medical Clinic III, St. Vincentius Hospital, Karlsruhe, Germany; 15 Medical Clinic I, University Hospital Mannheim, Mannheim, Germany; 16 Clinic for Cardiology, Ozypka Heart Center, Munich, Germany; 17 Clinic for Cardiology and Pneumology, University Hospital Göttingen, Göttingen, Germany; 18 Medical Clinic III, University Hospital Tübingen, Tübingen, Germany; 19 Clinic for Internal Medicine 2, Westpfalz-Clinic Kaiserslautern, Kaiserslautern, Germany; 20 Clinic for Internal Medicine III, Schwarzwald-Baar Hospital, Villingen-Schwenningen, Germany; 21 Medical Clinic B, Ludwigshafen Hospital, Ludwigshafen, Germany; 22 Medical Clinic IV, Municipal Clinical Center Karlsruhe, Karlsruhe, Germany; 23 Electrophysiology Bremen, Bremen, Germany; 24 Institute of Clinical Chemistry and Clinical Pharmacology, University Hospital Bonn, Bonn, Germany

**Keywords:** Oral anticoagulation, Atrial fibrillation, Pulmonary vein isolation, Cerebral microembolism, Stroke

## Abstract

**Background and objectives:**

Long-term oral anticoagulation (OAC) following successful catheter ablation of atrial fibrillation (AF) remains controversial. Prospective data are missing. The ODIn-AF study aimed to evaluate the effect of OAC on the incidence of silent cerebral embolic events and clinically relevant cardioembolic events in patients at intermediate to high risk for embolic events, free from AF after pulmonary vein isolation (PVI).

**Methods:**

This prospective, randomized, multicenter, open-label, blinded endpoint interventional trial enrolled patients who were scheduled for PVI to treat paroxysmal or persistent AF. Six months after PVI, AF-free patients were randomized to receive either continued OAC with dabigatran or no OAC. The primary endpoint was the incidence of new silent micro- and macro-embolic lesions detected on brain MRI at 12 months of follow-up compared to baseline. Safety analysis included bleedings, clinically evident cardioembolic, and serious adverse events (SAE).

**Results:**

Between 2015 and 2021, 200 patients were randomized into 2 study arms (on OAC: *n* = 99, off OAC: *n* = 101). There was no significant difference in the occurrence of new cerebral microlesions between the on OAC and off OAC arm [2 (2%) versus 0 (0%); *P* = 0.1517] after 12 months. MRI showed no new macro-embolic lesion, no clinical apparent strokes were present in both groups. SAE were more frequent in the OAC arm [on OAC *n* = 34 (31.8%), off OAC *n* = 18 (19.4%); P = 0.0460]; bleedings did not differ.

**Conclusion:**

Discontinuation of OAC after successful PVI was not found to be associated with an elevated risk of cerebral embolic events compared with continued OAC after a follow-up of 12 months.

**Graphical abstract:**

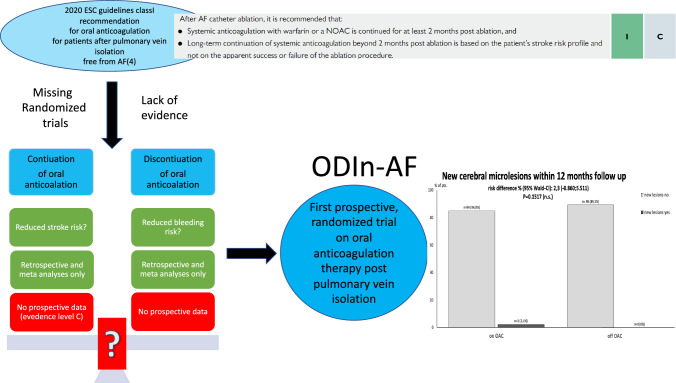

**Supplementary Information:**

The online version contains supplementary material available at 10.1007/s00392-023-02319-9.

## Introduction

Atrial fibrillation (AF) is the most common heart rhythm disorder. It affects approximately 4.5 million people in Europe [1]. AF strongly increases mortality, morbidity, and hospitalizations. Cardioembolic strokes represent the most severe complications related to AF [2], with AF being responsible for overall at least 20% of all ischemic strokes.

Oral anticoagulation (OAC) in AF patients significantly reduces the risk of stroke and death, and is the only therapeutic intervention that has repetitively shown to reduce mortality in patients with AF. Yet, OAC increases the risk of severe bleeding events [3]. The current guidelines recommend OAC in AF patients with relevant risk factors to reduce cardioembolic ischemic strokes [4]. For decades, vitamin K-antagonists (VKA) were the only substances available for OAC and have been shown to be effective in reducing the stroke risk in patients with AF [5]. VKA have major limitations, including multiple drug and dietary interactions and a very narrow therapeutic window.

Alternative anticoagulant treatments include recently developed oral thrombin inhibitors and oral factor Xa inhibitors. Among others, the phase III clinical trials investigating dabigatran proved the efficacy of non-vitamin K antagonist oral anticoagulants (NOAC) [6]. NOAC nowadays represent the established alternative for VKA in patients with non-valvular AF at embolic risk, and are recommended for initiation of OAC as first-line therapy in current guidelines [4].

Pulmonary vein isolation (PVI) is the cornerstone of catheter ablation for AF [7] representing the most successful treatment option for rhythm control [8]. Ablation of AF provides an improvement in quality of life [9] and reverses left ventricular dysfunction [10]. Recent data show that strict and early rhythm control reduces stroke risk and mortality [11]. Early PVI is the best treatment to achieve rhythm control [12]. Previous retrospective data show that the cardioembolic risk is relevantly reduced after successful PVI [13].

OAC should mandatorily be performed for 2–3 months after AF ablation procedures [4]. Continuation of OAC treatment after that period is controversial [13], even in the case of clinically successful catheter ablation; yet, current guidelines recommend the lifelong continuation of OAC in all these patients with a CHA_2_DS_2_VASc score > 1 (men) and > 2 (women), irrespective of evidence of recurrent AF (class of recommendation I, expert consensus) [4]. The net clinical benefit of OAC after successful AF ablation remains unclear, in particular in view of the risk of severe bleeding. The potential benefit for the individual patient who continues OAC after PVI is a risk reduction of clinically apparent strokes. The patients might additionally profit from an analogously reduced incidence of silent cardioembolic events. On the other hand, patients who discontinue OACs might benefit from the lower risk of potentially devastating bleeding events. There is insufficient prospective data supporting both assumptions and an urgent need for prospective data to clarify the best therapeutic strategy for these patients.

The ODIn-AF study is the first large-scale prospective study to systematically evaluate the necessity to continue OAC for prevention of silent cerebral lesions and clinical events in patients with paroxysmal or persistent AF followed-up for 12 months after successful PVI and with a relevant stroke risk, as assessed by a CHA_2_DS_2_VASc score ≥ 2.

## Methods

### Study design

The ODIn-AF study was a multicenter prospective, randomized, open-label, blinded endpoint (PROBE-design) interventional study. The details of the trial design have been published previously [14]. The study was approved by an independent ethics committee (057/15-AMG-ff), as well as the ethic boards of all institutions involved. ODIn-AF was conducted in accordance with the Declaration of Helsinki and the International Council for Harmonisation Good Clinical Practice Guidelines. ODIn-AF is registered with clinicalTrials.gov, NCT02067182; and EudraCT, 2013–003492-35. The ODIn-AF study was conducted at 22 experienced cardiology centers across Germany with > 300 catheter ablations/year.

Written informed consent was provided by all patients who participated. A Steering Committee adjudicated general study progress and study status, supported by the Clinical Study Core Unit, Study Center Bonn (SZB). An independent Data Safety Monitoring Board (DSMB) was established. It monitored the study progress and the safety of trial participants by reviewing all serious adverse events (SAE). DSMB further ensured the quality of the collected data. Selected endpoints were adjudicated by an independent Clinical Event Committee (CEC) comprising medical experts who were not involved in the study. The primary endpoint was adjudicated by an independent Radiology Core Lab (Dept. of Diagnostic and Interventional Radiology, University Hospital Bonn). Statistical analyses were independently performed by the SZB and Institute of Medical Biometry, Informatics and Epidemiology (IMBIE) of the University Hospital Bonn.

### Participants

The study included adult (> 18 years) patients scheduled for PVI (for paroxysmal or persistent (duration max. 12 months) AF and with an elevated stroke risk (CHA_2_DS_2_VASc score ≥ 2, resulting in an indication for OAC according to the current guidelines[4]). Patients free from AF episodes 6 months after successful antral PVI were randomized to the study arms. Inclusion and exclusion criteria were assessed at the timepoint of inclusion in the study and before randomization. Additionally, at the time of randomization, stable sinus rhythm (SR), assessed by 72-h Holter ECG and clinical evaluation, was confirmed.

### Inclusion and randomization

Following the baseline visit and written informed consent, patients were included in the trial before first antral PVI was performed. PVI was followed by 6-month run in period in which OAC had to be mandatorily performed (3 months blanking period + 3 months observation period). In case of AF recurrence in the observation period (i.e., months 4–6 after first PVI), repeat PVI was allowed to assure complete isolation of the pulmonary veins. The second ablation was followed again by 6-month run in (3 months blanking + 3 months observation). Patients were monitored during these periods for AF recurrences.

In case of clinical AF recurrence in the observation period, ECG documentation was mandatory before re-ablation or exclusion from the study. Pharmacological or electrical cardioversion and medical antiarrhythmic treatment were conducted according to current guidelines [4]. AF-free patients without clinical apparent or documented AF episodes during the observation periods after the first or the second PVI were randomized. To further prove freedom from AF in patients prior to randomization, a 72-h Holter ECG was analyzed. AF recurrence in the second observation period resulted in exclusion from the study.

For patients randomized to the OAC arm (on OAC with dabigatran 150 mg bid or reduced dose 110 mg bid in patients ≥ 80 years or on concomitant verapamil therapy), OAC with dabigatran was continued for 12 months after randomization, following recent guideline recommendations[4]. In patients in the second arm (off OAC), OAC was withdrawn after randomization; no placebo medication was administered (no masking).

### Trial investigations and procedures

All patients underwent antral PVI at least 6 to max. 7 months before randomization. Max. 7 days before randomization and after 12 months of follow-up, brain MRI was performed for evaluation of the primary endpoint.

#### PVI

All patients underwent transesophageal echocardiography prior to PVI to rule out left atrial thrombi. Radiofrequency (RF) and cryoballoon ablation are the most commonly used methods for antral PVI. The ablation techniques used in ODIn-AF followed the recommendations of the recent ESC guidelines on AF management [4]. Cooled RF or cryoballoon ablation were allowed at the local investigator`s discretion. The use of alternative or experimental ablation techniques or devices was not permitted.

### Magnetic resonance imaging (MRI)

Brain MRI performed at randomization and after 12 months of follow-up represent the central investigation for identification of the primary endpoint of the study. MRI scans were performed at a field strength ≥ 1.5 Tesla and included standard clinical transversal and coronal diffusion-weighted imaging (DWI), transversal T2-weighted turbo spin echo sequences, and transversal and coronal fluid attenuated inversion recovery (FLAIR) sequences.

All MRI studies were evaluated by a blinded core laboratory reading (Department of Diagnostic and Interventional Radiology, University Hospital Bonn). Scans were analyzed and reviewed by two independent, blinded, experienced radiologists in consensus for the presence, diameter, number, and vascular territory of new silent ischemic lesions.

### Visit investigations and follow-up

For the detection of predefined endpoints, all patients were followed in an intention-to-treat (ITT) manner for 12 months after randomization. The time schedule of the visits after randomization followed the accepted standard practice with on-site visits at 3, 9 and a final visit after 12 months [4]. All patients randomized remained in follow-up until completion of the final visit, death or withdrawal of consent.

Prior to first or second PVI, a 12-lead ECG and transesophageal echocardiography were performed. Procedural data were obtained during intervention. Randomization was performed after a total of 6 months after first or second PVI in the absence of clinical or documented AF recurrence. AF relapse after the first PVI encouraged second PVI followed again by 6 months before randomization. Patients with AF recurrence after second PVI were not randomized.

Max. 7 days prior to randomization, 72-h Holter ECG, 12-lead ECG, physical examination, clinically relevant AF symptoms, neurological examination, laboratory parameters, and brain MRI were conducted. Responses to the MOCA and QoL questionnaires were collected. At 3, 9, and during the final visit at 12 months, clinical history, 12-lead ECG, and 72-h Holter ECG, assessment of AF symptoms, laboratory parameters were evaluated. At the final visit 3 at 12 months, a brain MRI for the primary endpoint, a neurological examination, and the MOCA and QoL questionnaires were additionally performed.

Documented AF recurrences, assessed by AF-related symptoms and ECG recording in the off OAC arm, led to immediate initiation of OAC therapy with dabigatran. All patients were further followed-up in an ITT manner. AF episodes were defined in accordance to recent guidelines[4], detected in the performed 72-h Holter ECG or standard 12-lead ECG at regular follow-up visits or when patients were referred due to AF-related symptoms.

### Outcomes and adverse events

The primary endpoint of the ODIn-AF trial was the incidence of micro- and macro-embolic lesions including clinically silent lesions on brain MRI imaging up to 12 months after randomization. The primary endpoint was assessed after a 12-month period of study therapy. Missing data were considered as failure (i.e., occurrence of lesions was counted “positive” in such patients).

Secondary endpoints reported in this manuscript were incidence of clinically evident cardioembolic events (stroke, TIA, systemic embolism), bleeding events, hemorrhagic cerebral infarction, all-cause mortality, cardiovascular mortality neuropsychological evaluation (MOCA test), and quality of life (QoL EQ-5D score). These were assessed during the 12-month follow-up after randomization. Suppl. Table 1 shows the complete list of all secondary outcomes.

Safety analysis included all patients participating in the study. Life threatening, major and minor bleeding events, clinically evident cardioembolic events, and SAE were analyzed. SAE were defined as death, life-threatening, disabling events or events leading to or prolonging hospitalization. A complementary analysis of adverse events (AE) by severity of event and by relationship to trial treatment was performed. Safety data collection, documentation, and reporting of AE were performed according to the applicable German laws and regulations (AMG, GCP-V). Safety parameters were monitored by the DSMB. Safety events for group cross-overs were analyzed for the group the patient was allocated at end of individual trial participation.

In case of clinically relevant bleeding or cerebral ischemia events (TIA, stroke), patients were treated at local investigators´ discretions, following good clinical practice, recent guideline recommendations, and manufacturers´ recommendations.

The ITT population was defined as patients who were randomized and were analyzed as belonging to the treatment group according to randomized treatment assignment. The safety (SAF) population was defined as patients who were treated. For the SAF analysis, patients were analyzed as treated (rather than according to the randomization).

### Statistical analysis

As there are no reliable data existing on the long-term incidence of silent cerebral microembolism in patients after AF ablation, the expected risk was calculated based on published numbers for stroke (as a related outcome). For patients off OAC, the stroke risk associated with a CHA_2_DS_2_VASc score of 0 is estimated 0.78% per year. We expected a mean CHA_2_DS_2_VASc score of 2–3 in ODIn-AF, resulting in an expected stroke risk 5.3 times higher (4.2–4.5% per year)[3]. Recent guidelines suggest that after successful AF ablation, the risk of CE remains unchanged[3].

According to recent literature, the risk for silent CE in the overall population is 3% [15–17]. It was assumed that the increased risk of silent CE off OAC is comparable to the increased risk of apparent stroke assessed by the CHA_2_DS_2_VASc score, i.e., 5.3 times higher in the ODIn-AF study population (expected CHA_2_DS_2_VASc 2–3) versus a low-risk population (CHA_2_DS_2_VASc 0). This resulted in an estimated silent CE rate of 3% × 5.3 = 16% per year in patients off OAC after PVI. It has been shown [5] that OAC reduces the risk of silent CE by at least 50% in patients with paroxysmal AF. The risk was assumed at 8% annual silent CE rates for patients on OAC treatment (50% of the 16% CE rates for patients off OAC).

### Primary outcome analysis

The primary efficacy analysis was based on the occurrence of the primary endpoint at or before 12 months after the randomization visit 6 months after PVI. The rate of occurrence of micro- and macro-embolic lesions was compared between the treatment groups using a Cochran–Mantel–Haenszel test stratified for centers, at a type I error level of 5%. The primary analysis was done for the ITT population, which was defined as the set of patients who were randomized. Additionally, 95% confidence limits (Wald asymptotic and exact), were calculated for the difference in occurrence of lesions.

### Secondary outcome analyses

Predefined secondary endpoints were assessed during the 12 months of follow-up after randomization. Secondary endpoint analyses were descriptive; therefore, no formal statistical significance testing was performed. Comparisons between the treatment groups were assessed using Cochran–Mantel–Haenszel test stratified for centers.

### Safety analyses

Safety analysis included all patients who were treated. For the safety analysis, patients were analyzed as treated (rather than according to the randomization). A complementary analysis of AE by severity of event and by relationship to trial treatment was performed. Fisher’s exact and Chi-square tests were used to compare incidences of AE. Laboratory parameters were analyzed descriptively.

## Results

**Fig. 1:**
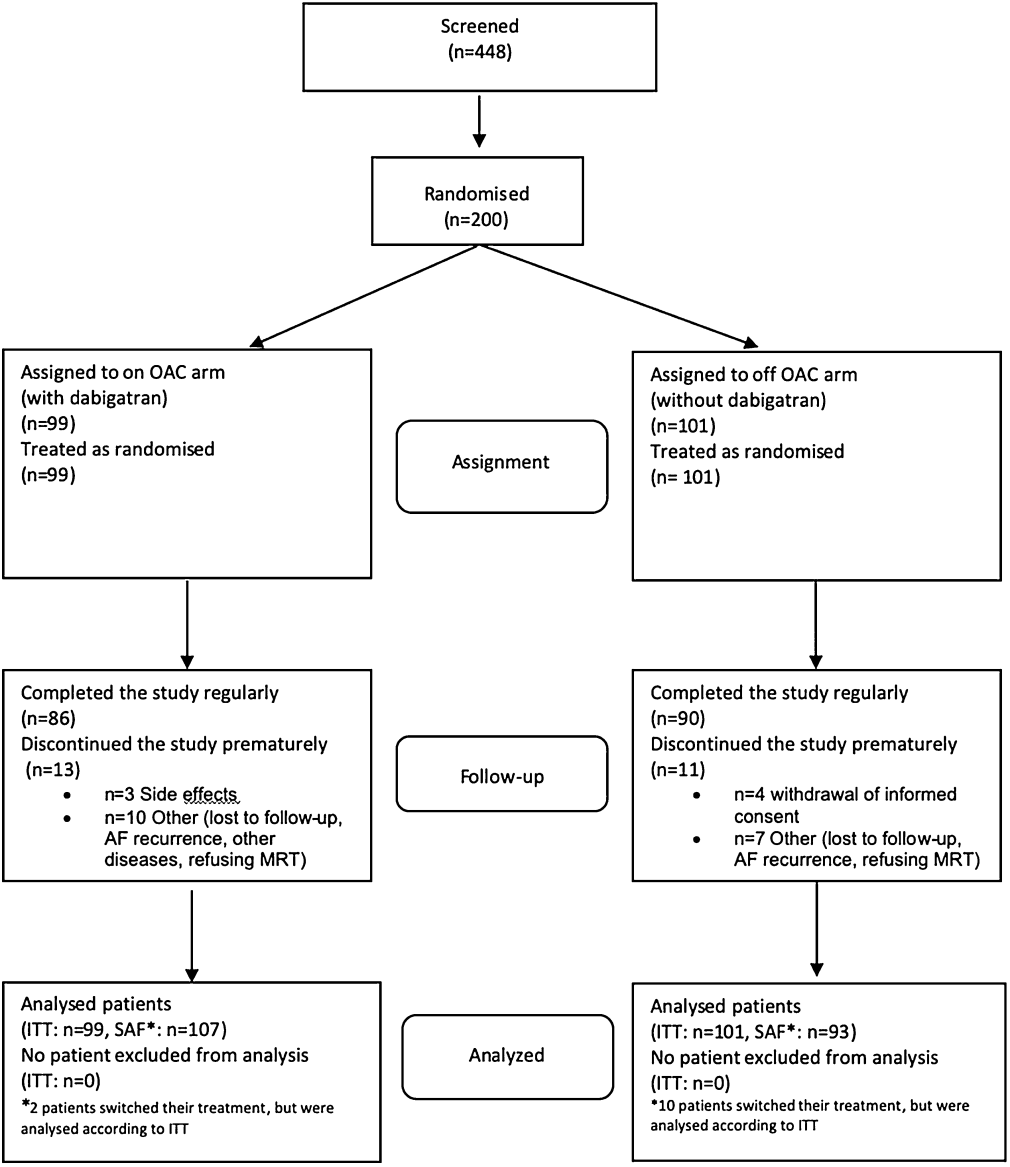
Trial profile. Of the 448 patients screened, 200 underwent inclusion, first or second PVI and had no AF recurrences in the 3-month observation period prior to randomization. Patients were randomized to one of the treatment arms. All patients who underwent randomization were included in the primary ITT analysis. ITT: intention to treat; OAC: oral anticoagulation; SAF: safety analysis

A total of 448 patients were screened for the study across 22 centers in Germany between September 9th 2015 and September 15th 2021. Screening included medical history taking and verification of inclusion and exclusion criteria. Main reasons for screening failure were refusal of study participation by the patient, concomitant diseases such as hyperthyroidism or severe heart failure, need for extensive ablation strategies due to concomitant atrial flutter or ectopic tachycardia, and CHA_2_DS_2_VASc score lower than 2. No selection bias was expected by these screening exclusion and a total of 200 patients underwent PVI for AF (66% paroxysmal; 34% persistent) followed by the run in phase and were randomized to one of the two study arms (with dabigatran: OAC *n* = 99; without OAC: off OAC *n* = 101) (Fig. 1). The number of subjects completing the study, providing assessments 12 months after randomization, was comparable in both arms [OAC *n* = 87 (87.9%), off OAC *n* = 91 (90.1%)]. Demographics and baseline characteristics were well balanced between the study arms (Table 1).

**Table 1:** Demographic data and clinical characteristics of the patients at baseline

Characteristic	Total (*n* = 200)	On OAC (*n* = 99)	Off OAC (*n* = 101)
Age, years	67.2 ± 7.5	67.3 ± 7.2	67.1 ± 7.7
Female, no. (%)	88 (44.0)	43 (43.4)	45 (44.6)
BMI, kg/m^2^	29.3 ± 5.5	28.7 ± 6.1	29.8 ± 4.8
*Type of AF, no. (%)*			
Paroxysmal	133 (66.5)	65 (65.7)	68 (67.3)
Persistent (max. 12 mths.)	63 (31.5)	30 (30.3)	33 (32.7)
SR (inclusion ECG), no. (%)	135 (67.5)	67 (67.7)	68 (67.3)
Heart rate—bpm (inclusion ECG)	70.7 ± 21.7	71.3 ± 20.7	70.0 ± 22.7
LVEF, %	57.8 ± 9.0	58.0 ± 9.4	57.7 ± 8.6
Left atrial volume, ml	63.4 ± 27.4	60.1 ± 22.1	66.5 ± 31.4
CHA_2_DS_2_VASc score^b^	2.6 ± 0.8	2.6 ± 0.8	2.6 ± 0.7
CHA_2_DS_2_VASc score, no. (%)			
2	109 (54.5)	58 (58.6)	51 (50.5)
3	60 (30.0)	25 (25.3)	35 (34.7)
4	28 (14.0)	13 (13.1)	15 (14.9)
5	3 (1.5)	3 (3.0)	0 (0.0)
HasBled score	1.4 ± 0.7	1.3 ± 0.7	1.5 ± 0.7
HasBled score, no. (%)			
0	10 (5.0)	7 (7.1)	3 (3.0)
1	52 (26.0)	27 (27.3)	25 (24.8)
2	51 (25.5)	23 (23.2)	28 (27.7)
3	4 (2.0)	2 (2.0)	2 (2.0)
EHRA symptoms score, no. (%)			
I	6 (3.0)	0 (0.0)	6 (5.9)
II	63 (31.5)	30 (30.3)	33 (32.7)
III	64 (32.0)	31 (31.3)	33 (32.7)
IV	3 (1.5)	2 (2.0)	1 (1.0)
NYHA score, no. (%)			
I	39 (19.5)	18 (18.2)	21 (20.8)
II	60 (30.0)	30 (30.3)	30 (29.7)
III	21 (10.5)	13 (13.1)	8 (7.9)
IV	1 (0.5)	0 (0.0)	1 (1.0)
Prior ineffective AA, no. (%)			
0	77 (38.5)	40 (40.4)	37 (36.6)
1	98 (49.0)	48 (48.5)	50 (49.5)
2	12 (6.0)	6 (6.1)	6 (5.9)
*Concomitant cardiovascular diseases*			
Arterial hypertension, no. (%)	175 (87.5)	86 (86.9)	89 (88.1)
CKD (GFR < 60 ml/min), no. (%)	14 (7.0)	6 (6.1)	8 (7.9)
Diabetes mellitus, no. (%)	41 (20.5)	21 (21.2)	20 (19.8)
Peripheral arterial disease, no. (%)	5 (2.5)	4 (4.0)	1 (1.0)
CAD, no. (%)	36 (18.0)	16 (16.2)	20 (19.8)
Stable heart failure, no. (%)	23 (11.5)	12 (12.1)	11 (10.9)
Medication at inclusion, no. (%)^c^			
NOAC	149 (74.5)	73 (73.7)	76 (75.2)
VKA	22 (11.0)	10 (10.1)	12 (11.9)
Platelet inhibitor	7 (3.5)	1 ( 1.0)	6 (5.9)
Class 1 AA	17 (8.5)	5 (5.1)	12 (11.9)
Class III AA	24 (12.0)	11 (11.1)	13 (12.9)
Beta-blocker	162 (81.0)	78 (78.8)	84 (83.2)
Calcium antagonist	51 (25.5)	24 (24.2)	27 (26.7)
Digitalis glycoside	4 (2.0)	3 (3.0)	1 (1.0)
ACE inhibitor	72 (36.0)	37 (37.4)	35 (34.7)
ATII receptor antagonist	63 (31.)	32 (32.3)	31 (30.7)
Diuretic	73 (36.5)	40 (40.4)	33 (32.7)
Statin	76 (38.0)	32 (32.3)	44 (43.6)
*PVI*			
First intervention, no. (%)			
RF	50 (25.0)	27 (26.2)	23 (22.8)
Cryo	150 (75.0)	72 (73.8)	78 (77.2)
Additional CTI ablation	26 (13.0)	10 (10.1)	16 (15.8)
Second intervention, no. (%)	*n* = 6	*n* = 2	*n* = 4
RF	4 (66.6)	1 (50)	3 (75)
Cryo	2 (33.3)	1 (50)	1 (25)

The mean CHA_2_DS_2_VASc score in all patients was 2.6 ± 0.8, ranging between 2 (54.5%) and 5 (1.5%); the mean HasBled score was 1.4 ± 0.7, defining a population at high stroke risk and moderate bleeding risk. No differences were found regarding CHA_2_DS_2_VASc and HasBled scores between the treatment groups (Table 1).

Continuous variables are summarized by mean ± standard deviations. The randomized treatment groups did not show differences and were well balanced.

*AA* antiarrhythmic agent, *AF* atrial fibrillation, *ACE* angiotensin-converting enzyme, *AT* angiotensin, *BMI* body mass index, *CAD* coronary artery disease, *CKD* chronic kidney disease, *Cryo* cryoballoon ablation, *CTI* cavotricuspid isthmus, *LVEF* left ventricular ejection fraction, *NOAC* non-vitamin K antagonist oral anticoagulant, *OAC* oral anticoagulation, *PVI* pulmonary vein isolation, *RF* radiofrequency ablation, *VKA* vitamin K antagonist.

^a^Stable heart failure is defined as New York Heart Association (NYHA) stage II or a LVEF of less than 50%

^b^CHA_2_DS_2_VASc score (an assessment of the risk of stroke among patients with atrial fibrillation) ranges between 0 and 9, with higher scores indicating a higher risk of stroke.

^c^The absence of symptoms is defined as a European Heart Rhythm Association (EHRA) score of I. The EHRA score groups symptoms related to AF into four classes from I (asymptomatic) to IV (severe symptoms at rest).

After inclusion, first antral PVI was conducted in all patients. One hundred fifty patients (75%) were treated with cryoballoon ablation, fifty patients (25%) were treated with point-by-point RF ablation, 13% received additional RF ablation of the cavotricuspid isthmus for typical atrial flutter. In case of AF recurrence in the 3-month observation period of the run in phase, second ablation was conducted in a total of six patients (2 patients received cryoballoon ablation, four patients received RF), followed by a second run in phase. No differences were seen in ablation type or need for secondary ablation in the patients randomized to OAC versus off OAC arm. Use of antiarrhythmic drugs (Class I and Class III) prior to was 20.5% in the study population and was reduced to 4.3% at final visit 3. At 12 months, the drop-out rates were comparable (12.1% in the OAC and 9.9% in the off OAC group; Fig. 1).

In the patients treated with dabigatran, the compliance to therapy was rated good in 70.7% of the patients receiving OAC at visit 3 (Suppl. Table 2). Two patients at close out visit refused 72-h Holter ECG. All other patients received 72-h Holter ECGs protocol conform at 3-, 9-, and 12-month FU visits with mean recording durations of > 69 h for all visits in both groups. During the 12-month follow-up, ten patients from off OAC group started treatment with OAC, mainly due to documented AF recurrences, that triggered protocol compliant allocation to OAC. Two patients from OAC group stopped their treatment because of relevant gastrointestinal side effects of the OAC. No differences were present in these cross-overs compared to these patients adherent to randomized treatments (non-cross-overs), regarding demographical or clinical characteristics. Clinical reasons for switching treatment are depicted in Suppl. Table 3. AF recurrences were equally distributed across the groups and occurred in 9% in the OAC arm versus 7.9% in the off OAC arm.

**Fig. 2:**
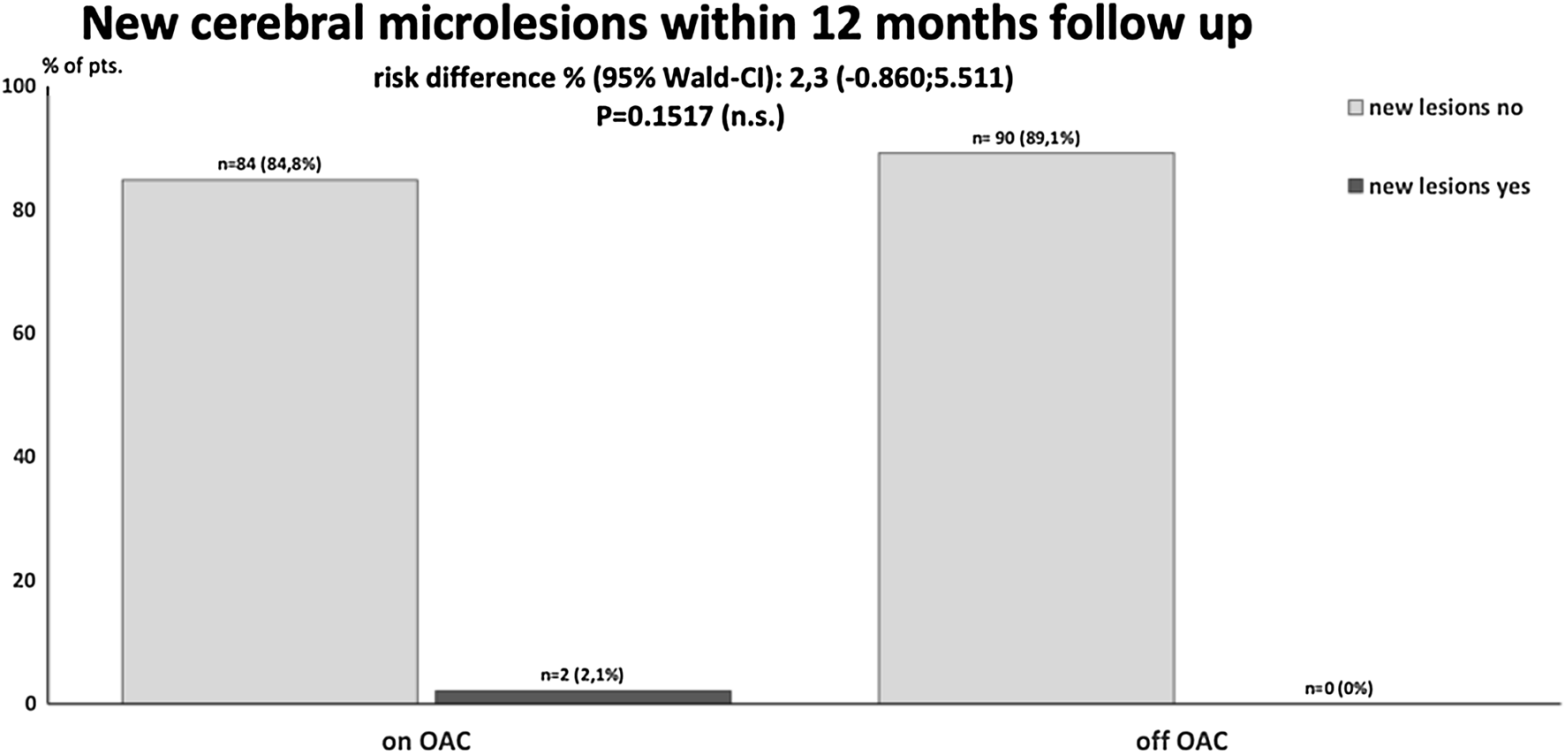
ITT analysis of the primary outcome. The Cochran–Mantel–Haenszel test at the two sided of 5% type I error level was used for analysis of the primary outcome. There were no significant differences in the primary outcome between the treatment groups. Thirteen patients (13.1%) in the OAC and eleven patients in the off OAC group did not receive final MRI investigation. Missing values were ignored. Pts.: patients; missing: missing final MR data

On baseline MRI, just one patient randomized to OAC showed cerebral microlesions. Regarding the ITT analysis, the primary endpoint (occurrence of new micro- and macro-embolic lesions up to 12 months after randomization), two patients (2%) on OAC showed new cerebral microlesions and no patient (0%) off OAC, ****P = 0.1517; 95%-CI:-0.860%;5.5711% (Table 2; Fig. 2). The two patients with microembolisms were male, 65 and 67 years of age and both had a CHA_2_DS_2_VASc score of 2. Thirteen patients in the OAC group and eleven in the off OAC group did not receive cerebral MRI at close out, i.e., due lost to FU, withdrawal of consent or declined MR examination. As predefined, analysis for the primary endpoint was also performed with these missing data considered as failure, and thus counted as occurrence of lesions. Again, there was no significant difference between the OAC and off OAC arm (15.2% versus 10.9%; P = 0.4545, 95% CI:-5.056%;13.577%). No macro-embolic lesion appeared on MRI in the treatment groups.

**Table 2:** Efficacy outcomes

Outcome	Total (*n* = 200)	On OAC (*n* = 99)	Off OAC (*n* = 101)	*P* value On vs. off
*Primary endpoint*				
Incidence of new micro- and macro-embolic lesions on MRI after 1y FU compared to baseline MRI^a^, no. (%)	2 (1.0)	2 (2)	0 (0)	0.1517
*Secondary endpoints*				
Clinical cardioembolic events, no. (%) (stroke, TIA, systemic embolism)	0	0	0	n.a
Hemorrhagic cerebral infarction, no. (%)	0	0	0	n.a
Other thrombotic or thromboembolic events (MI, DVT, PE), no. (%)	0	0	0	n.a
Bleedings^b^ (life-threatening/major/minor), no. (%)	3 (1.5)	2 (2.0)	1 (1.0)	0.4738
All-cause mortality	0	0	0	n.a
Cardiovascular mortality	0	0	0	n.a
Change in neuropsychological evaluation (MOCA test)		1.2x ± 5.2	0.8 ± 3.2	0.5145
Change in quality of life (EQ-5D score)		3.0 ± 20.7	− 0.7 ± 16.9	0.1888

Continuous variables are summarized by mean ± standard deviations. Analyses were performed using Student’s *t* test for continuous and Cochran–Mantel–Haenszel for categorical variables. There were no significant differences in the primary and the secondary outcomes between the treatment groups.

*DVT* deep vein thrombosis, *FU* follow-up, *MI* myocardial infarction, *MRI* magnetic resonance imaging, *OAC* oral anticoagulation, *PE* pulmonary embolism, *TIA* transient ischemic attack.

^a^As predefined for the primary endpoint, missing values were counted as occurrence of cerebral embolism.

^b^All observed bleedings that occurred were graded minor.

**Fig. 3:**
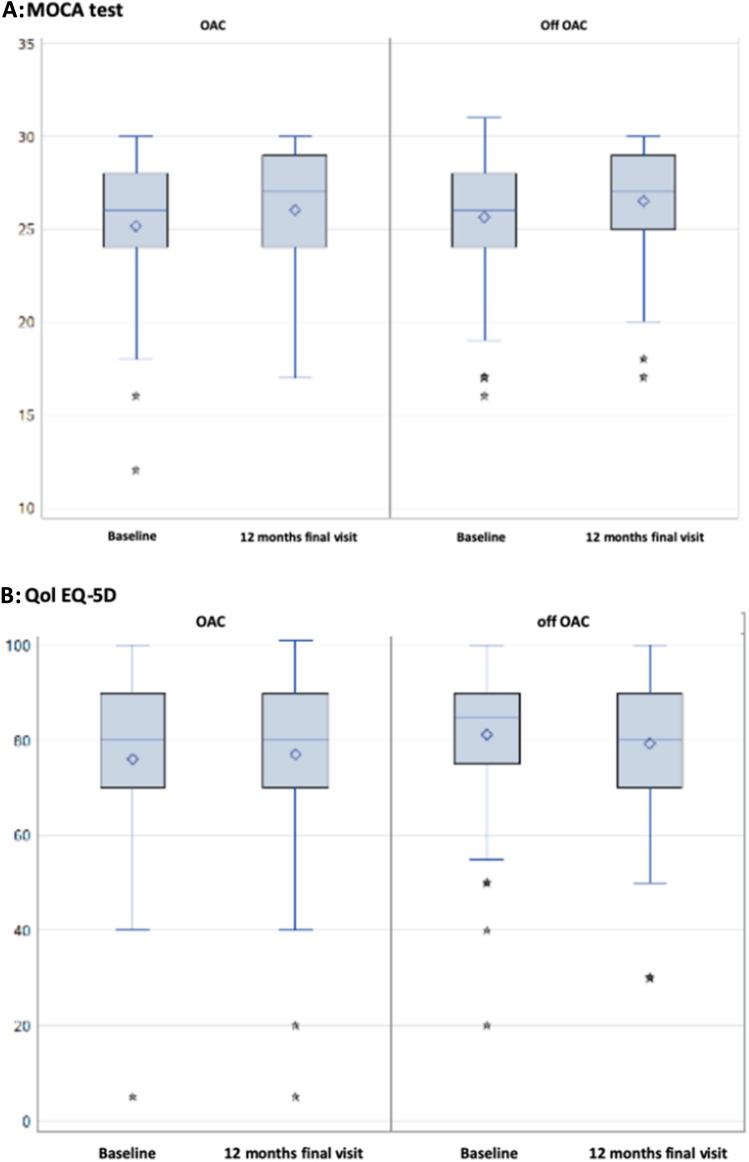
Neuropsychological/neurocognitive assessment and quality of life. All values are presented as mean ± standard deviation of the mean (SD). Analyses were performed using Student’s *t* test. No significant differences were found. *BL* baseline, *OAC* oral anticoagulation, *QoL* quality of life. **A** The neuropsychological evaluation and assessment of neurocognitive deficits was performed using the Montreal cognitive assessment questionnaire (MOCA test). The score ranges between 0 and 30, with lower scores indicating worse cognitive function. At least mild cognitive impairment is defined MOCA score of less than 26. **B** Quality of life (QoL) was assessed using the QoL questionnaire EQ-5D. The score ranges between 0 and 100, with lower scores indicating worse QoL

Secondary outcomes were analyzed by ITT. No clinically apparent cardioembolic event or stroke occurred in the groups in the 12 months of follow-up. No patient died during the trial. No differences were found between the OAC and the off OAC group in any of the other secondary endpoints (Table 2). Relevant bleedings did not differ among the groups (Table 2). The use of antiplatelet therapy was equal between the groups during FU (OAC: 0%; off OAC: 4.3%). The changes from baseline of the neuropsychological test (MOCA) and quality of life (QoL EQ-5D score) were not found to differ between the groups (Fig. 3).

Safety results were analyzed in the predefined safety population (Table 3) different to ITT populations, the ten patients from off OAC who switched their treatment to OAC were considered to be allocated to the OAC group; the two patients that switched from OAC that discontinued treatment were considered to be allocated to the off OAC group. Adverse events were reported by a total of 108 patients [OAC *n* = 72 (67.3%), off OAC *n* = 46 (49.5%); P = 0.011]. Nine patients [OAC *n* = 6 (5.6%), off OAC *n* = 3 (3.2%); P = 0.5080] reported drug-related AEs. The percentage of patients with SAEs (52 in total) was higher in the OAC arm [OAC: *n* = 34 (31.8%); off OAC: *n* = 18 (19.4%); P = 0.046]. Cardiac disorders represented the most common AEs and were significantly elevated in the patient group with OAC (OAC: 35.5%, off OAC: 10.8%; P < 0.0001), mainly triggered by arrhythmia recurrences.

**Table 3:** Safety outcomes

Safety outcome	Total (*n* = 200)	On OAC (*n* = 107)	Off OAC (*n* = 93)	*P* value On vs. off
Any adverse event	118 (59)	72 (67.3)	46 (49.5)	0.0106
Drug related adverse event	9 (4.5)	6 (5.6)	3 (3.2)	0.5078
Severe adverse event	18 (9.0)	10 (9.3)	8 (8.6)	0.8546
*Predefined safety endpoints*				
All bleedings, no. (%)	3 (1.5)	2 (2.0)	1 (1.0)	0.4738
Life-threatening	0	0	0	n.a
Major	0	0	0	n.a
Minor	3 (1.5)	2 (2.0)	1 (1.0)	0.4738
Stroke and clinical evident embolic events	0	0	0	n.a
*Serious adverse events*				
Total	52 (26.0)	34 (31.8)	18 (19.4)	0.0458
Atrial fibrillation	12 (6.0%)	10 (9.3%)	2 (2.2%)	0.0384
Atrial flutter	3 (1.5%)	3 (2.8%)	0 (0.0%)	0.2499
Palpitations	2 (1.0%)	2 (1.9%)	0 (0.0%)	0.4999
Heart failure	2 (1.0%)	2 (1.9%)	0 (0.0%)	0.4999
Surgery	2 (1.0%)	2 (1.9%)	0 (0.0%)	0.4999
Inguinal hernia	2 (1.0%)	2 (1.9%)	0 (0.0%)	0.4999
Prostate cancer	2 (1.0%)	1 (0.9%)	1 (1.1%)	1.0000
Osteoarthritis	2 (1.0%)	1 (0.9%)	1 (1.1%)	1.0000

Note that patients could have had more than one event, and therefore the potential total sum of events is higher than the number of patients with events. The Cochran–Mantel–Haenszel, Fisher’s exact, and Chi-square tests were used to compare the groups

*OAC* oral anticoagulation.

## Discussion

AF is an independent risk factor for cardioembolic events and increases the stroke risk by almost fivefold [18]. Cerebral microembolisms represent an independent risk factor for the development of later clinically apparent cerebral embolic events [19, 20] and were, therefore, used as subclinical surrogate for the risk of clinical apparent cerebral embolic events. Medication with OAC (VKA and NOAC) according to the CHADS_2_ and CHA_2_DS_2_VASc scores reduces stroke risk and improves survival in AF patients [11]. Current guidelines [4] suggest long-term OAC after successful catheter ablation for AF, although prospective data are lacking and even in the era of NOAC, bleeding complications remain a main cause of morbidity and mortality.

ODIn-AF is the first randomized, prospective study to evaluate the effect of OAC on the 12 months incidence of silent cerebral embolic infarcts in patients with an elevated of cardioembolic events, but free from AF after successful PVI. There was no significant difference found in the patient groups on and off OAC. Incidences of cerebral micro-insults were low in both groups but no excess microembolism was present in the patients off OAC. Despite a CHA_2_DS_2_VASc score ≥ 2 in both groups, clinically significant embolisms and strokes were not found in secondary endpoint and safety analyses. Bleeding events under OAC with dabigatran were very low, and lower than the bleeding risk estimable from the RE-LY study. Here, a major bleeding event rate of 3.32% per year was found under OAC (150 mg dabigatran bid) [8] and life-threatening bleeding occurred in up to 1.45%. Cessation of OAC was not associated to better quality of life in the patient group off OAC. OAC therapy seems, thus, to be generally well accepted in the investigated on OAC group.

The predictive values of CHADS_2_ and CHA_2_DS_2_VASc scores were previously investigated in patients after AF catheter ablation [21]. Both scores remained valid predictors of thromboembolic events after ablation in particular in patients with and without AF recurrences after ablation, with recurrence of AF representing an independent predictor of embolism. Elimination of AF, thus, seems the most important factor for freedom from embolic events. In ODIn-AF, only patients proven to be free from AF by routine clinical assessment 6 months after catheter ablation of AF were randomized. ODIn-AF followed a feasible and generally conductible follow-up protocol for AF recurrences, with regular 72-h Holter ECG and patient contact in case of clinically apparent AF relapse or palpitations. The aim of this screening protocol (rather than continuous monitoring by implantable loop recorders) for AF recurrences was to establish an easily executable follow-up in general practice.

The expert consensus of current guidelines advises to continue OAC in patients with elevated stroke risk independent from detected AF recurrence, as stroke rate and rate of silent embolisms may persistently be elevated due to asymptomatic AF relapses. Current recommendations, therefore, put patients on potential bleeding risk while hoping to prevent embolic events, yet in the absence of prospective data supporting this strategy. The results of a retrospective cohort study showed that discontinuation of warfarin treatment after PVI was associated to elevated risk of cardioembolic events, especially those who have previously experienced an ischemic stroke [22]. In ODIn-AF, history of previous stroke was, therefore, an exclusion criteria.

Two large-scale, but retrospective, studies showed that up to 25% of patients with a CHA_2_DS_2_VASc score ≥ 2 discontinued OAC after AF ablation [23, 24]. The clinical impact of this frequent violation of recommended OAC therapy by patients and/or physicians remained unclear. Liu and co-workers showed in a large-scale meta-analysis of prospective observational studies that discontinuation of OAC after successful AF ablation is safe and observed an increased risk of major bleeding in patients remaining on OAC [25]. In accordance to that, the ODIn-AF study suggests that cessation of OAC after AF ablation in the absence of AF might be a safe strategy in such patients.

It is questionable if the patient population after successful AF ablation and without clinical or ECG-documented AF recurrences exhibits the same risk for cardioembolic events as non-ablation patients. An analysis including 20 studies and > 20,000 patients showed elevated embolic risk after AF ablation for patients off OAC. The study designs and endpoints of these mainly retrospective analyses were heterogenous [26]. In a large retrospective analysis involving 37.908 patients [20], catheter ablation reduced the rate of cerebrovascular incidents by 53% at 12 months and by 41% during long-term follow-up. The stroke rates in AF patients with ablation were similar to a healthy, matched control population without documented AF. These results pointed toward a different stroke risk after successful AF ablation in patient populations free from AF recurrences.

Supporting this, data from an international study group compared the outcome of a large cohort of 1273 patients after catheter ablation to medically treated patients from the EURO Heart survey [27]. Freedom from AF was the strongest independent predictor of stroke-free survival. In this study cohort, OAC was stopped in 809 patients (64%) after ablation. Despite a CHADS_2_ score of 0, 1, 2, and 3 or higher, the annual stroke rate was only 0.3%, 0%, 0.7%, and 0%, respectively, in these patients. Numerous retrospective, non-randomized trials further investigated OAC management and discontinuation of OAC after AF catheter ablation [28, 29]. Several meta-analyses were conducted evaluating cessation of OAC after AF ablation [24, 30]. The largest systematic review of 16 cohort studies (> 25,000 patients) compared embolic events in patients with and without OAC after AF ablation. Even after stratification for CHADS_2_ and CHA_2_DS_2_VASc score, no differences were found regarding cardioembolic events between the groups [13]. Heterogeneity of the analyzed studies, low NOAC use, and the fact that patients with cessation of OAC had tendentially lower CHADS_2_ and CHA_2_DS_2_VASc scores in some studies were potential confounders.

In conclusion, these inhomogeneous, retrospective analyses suggested that cessation of OAC after AF ablation could be safe and may lower bleeding risk. On the other hand, other studies, yet with obvious limitations, suggested potentially elevated risk in such patients [28]. In the light of these inconclusive results, randomized clinical trials were strongly encouraged [31], and ODIn-AF is the first study to show feasibility of cessation in a defined population of patients with CHA_2_DS_2_VASc score ≥ 2 and AF-free after PVI. Increasing number of risk factors with time may elevate stroke risk after PVI. In a study of 14,606 AF patients not on OAC with a CHA_2_DS_2_VASc score <  = 1, 49% of patients acquired at least one new risk factor over 4 years. It is, thus, mandatory to reassess stroke risk in patients off OAC on a regular base [32]. Kaplan et al. evaluated cardioembolic risk depending on AF duration and CHA_2_DS_2_VASc score in > 21,000 patients with implantable devices off OAC. The investigators showed that the stroke risk with a CHA_2_DS_2_VASc score 0 and 1 is low, and that with increasing CHA_2_DS_2_VASc score ≥ 2 cardioembolic risk rises inversely proportional to documented AF-episode durations. The higher the CHA_2_DS_2_VASc score, the shorter AF episodes seem to provoke cardioembolic events. AF in patients with CHA_2_DS_2_VASc score ≥ 2 may, thus, just be a coexistent factor for other vascular morbidities associated to AF, leading to additionally elevated stroke risk [33]. We did not show elevated thromboembolic events in patients off OAC with a median CHA_2_DS_2_VASc score of 2 after AF ablation. In ODIn-AF, regular Holter ECGs and clinical presentation/symptoms and validation by CEC were utilized for screening for AF recurrences. Yet, 10% of patients were identified in the off OAC group with AF recurrence and switched to OAC therapy OAC arm. None of the cross-overs showed relevant events regarding primary and secondary endpoints, pointing toward the fact that the in ambulatory setting, easy-to-apply Holter ECGs rather than continuous monitoring and clinical evaluation seem sufficient to monitor these patients and to safely change therapy to OAC, when AF relapses are present.

### Limitations

As dabigatran was used exclusively, no general conclusions can be drawn for other NOACs available, but it is likely from the approval studies and clinical experience that the results might be generally applicable to all NOAC.

Patients in ODIn-AF were followed-up for 12 months. Although it has been shown that most recurrences of AF appear in this time period after AF ablation [34], no conclusions can be drawn regarding longer follow-up. As patients may develop recurrences after 2 years, and later [32], ODIn-AF should encourage larger prospective studies with longer follow-up periods. OAC was started in ODIn-AF in all patients off OAC, when AF recurrences occurred. Asymptomatic AF episodes were potentially overseen, but there was no clinical or MR morphological substrate for potential higher stroke risk due to asymptomatic and/or non-recognized AF episodes in the off OAC group, admittedly in a limited number of patients. As overall low bleeding rates were present in both study groups, an expected safety benefit regarding such events in patients off OAC could not be shown in the ODIn-AF 12-month follow-up, but relevantly more SAE were present in the patients treated with OAC.

No continuous heart rate monitoring was used in ODIn-AF, which might lead to underestimation of AF relapses. ODIn-AF aimed to use a feasible and screening protocol (rather than continuous monitoring by implantable loop recorders) for AF recurrences to investigate the effect of AF ablation on cardioembolic events, an easily executable follow-up for general practice. Perspectively, use of smart watches and external devices will facilitate heart rate monitoring for individual patients and might be a strategy for further studies on this topic.

When ODIn-AF was started, general guidelines advised lifelong OAC as a Class I recommendation in all patients with a CHA_2_DS_2_VASc score ≥ 2. In the following guidelines[4], female gender as a risk factor was relativized and downgraded, resulting in a Class I recommendation for OAC in women with a CHA_2_DS_2_VASc score elevated ≥ 3. In the ODIn-AF study, inclusion criterion was ≥ 2 for all patients regardless of gender. Therefore, during the study course, OAC indication for female patients with a CHA_2_DS_2_VASc score of 2 was a Class IIa recommendation (female gender and one risk factor). As the mean CHA_2_DS_2_VASc score in the ODIn-AF study was 2.6 ± 0.8, the trial results, therefore, account for a higher risk male population, as compared to a lower risk female population.

ODIn-AF excluded patients with prior stroke, as these represent a very high-risk population for recurrent embolism [35]. The highest CHA_2_DS_2_VASc score was 5. This has to be considered, when clinical decisions regarding discontinuation of OAC after AF ablation are made, as very high-risk patients were underrepresented.

Due to the relatively small patient number, larger prospective studies will be needed to confirm the promising results of ODIn-AF.

## Conclusion

ODIn-AF is, thus, the first randomized study that evaluated the benefit of long-term NOAC therapy after effective AF ablation compared to cessation of NOAC 6 months after. No difference in regard to asymptomatic cerebral infarcts were detected. The results of ODIn-AF encourage larger scale randomized trials to further confirm the safety of withdrawal of OAC after effective ablation of AF.

### Outlook

The consistency of the ODIn-AF results with retrospective data from numerous studies and meta-analyses suggests that discontinuation of OAC in patients without AF recurrence after PVI may not be associated with an increased incidence of cardioembolic events. These results encourage further larger scale randomized clinical trials.

### Supplementary Information

Below is the link to the electronic supplementary material.

Supplementary file1 (DOCX 22 KB)
