# Supplementary material for: Prevention of cerebral thromboembolism by oral anticoagulation with dabigatran after pulmonary vein isolation for atrial fibrillation: the ODIn-AF trial
Source: Clin Res Cardiol. 2023 Nov 3;113(8):1183–99. doi: 10.1007/s00392-023-02319-9 (PMC11269394; doi:10.1007/s00392-023-02319-9)
Supplement: Supplementary file 1 — Supplementary file1 (DOCX 22 KB) [file 392_2023_2319_MOESM1_ESM.docx]

**Supplementary Material**

***Suppl. Table1: Secondary endpoints of the ODIn-AF study***

| 1. Localisation, size and number of new micro- and macro-embolic lesions on cerebral MRI |
| --- |
| 1. Incidence of clinically evident cardioembolic events (stroke, TIA, systemic embolism) |
| 1. Incidence of clinically apparent neurological deficits |
| 1. Severeness of neurological deficits (modified ranking severity scale (mRS)) |
| 1. Incidence of other thrombotic or thrombo-embolic events (myocardial infarction, deep vein thrombosis, pulmonary embolism) |
| 1. Life-threatening / major / minor bleedings |
| 1. Hemorrhagic cerebral infarction |
| 1. All-cause mortality |
| 1. Cardiovascular mortality |
| 1. Correlation of cardio-embolic events with method used for PVI (cryo-balloon versus radiofrequency ablation) |
| 1. Correlation of cardio-embolic events with arrhythmia recurrence (AF or atrial flutter post ablationem with ECG documentation or symptoms) |
| 1. Correlation of cardio-embolic events with echocardiographic parameters (i.e. left atrial size, left ventricular function, left atrial appendage velocities) |
| 1. Quality of life questionnaire (EQ-5D) |
| 1. Neuropsychological questionnaire and assessment of neurocognitive deficits (Montreal cognitive assessment Questionnaire (MOCA test)) |

***Suppl.Table2: Compliance by category in the ITT population***

|  | | | *Group* | |
| --- | --- | --- | --- | --- |
| *Visit* |  | Compliance category (n (%) | *(Experimental; on OAC) n=99* | *(Control; no OAC) n=101* |
| Visit 1 (3 months after randomisation) |  | missing | 3 (3.0%) | 101 (100.0%) |
|  |  | good | 89 (89.9%) | 0 (0.0%) |
|  |  | moderate | 3 (3.0%) | 0 (0.0%) |
|  |  | poor | 4 (4.0%) | 0 (0.0%) |
| Visit 2 (9 months after randomisation) |  | missing | 10 (10.1%) | 98 (97.0%) |
|  |  | good | 76 (76.8%) | 1 (1.0%) |
|  |  | moderate | 7 (7.1%) | 0 (0.0%) |
|  |  | poor | 6 (6.1%) | 2 (2.0%) |
| Visit 3 (12 months after randomisation) |  | missing | 15 (15.2%) | 96 (95.0%) |
|  |  | good | 70 (70.7%) | 4 (4.0%) |
|  |  | moderate | 13 (13.1%) | 1 (1.0%) |
|  |  | poor | 1 (1.0%) | 0 (0.0%) |
| ________________________________________________________________________________________________________________________ Percentage based on patients in the ITT population good - > 80%, poor - < 50%, moderate - neither good nor poor | | | | |

The compliance C_i_ for patient i was computed according to:

$$C_{i}=\frac{D_{i}^{t}*100}{D_{i}^{p}}$$

where $D_{i}^{p}$ is the total number of pills foreseen to patient i and $D_{i}^{t}$ is the total number of pills actually taken by the patient during the study for patient i. OAC: oral anticoagulation.

***Suppl. Table3: Adverse events for group switchers - Intensity, seriousness, relationship. Population: randomized***

| *Patient in group* | *AE no.* | *AE*  *diagnosis* | *Severity* | *SAE* | *SAE-criteria* | *relationship to study drug* | *Change*  *in study drug* |
| --- | --- | --- | --- | --- | --- | --- | --- |
| experimental; on OAC | 1 | Gastralgia | mild | no | . | yes | permanently discontinued |
|  | 2 | Exanthema | severe | yes | hospitalisation | no | . |
| experimental; on OAC | 1 | Cervical spine pain | moderate | no | . | no | . |
|  | 2 | Gastralgia | mild | no | . | yes | permanently discontinued |
|  | 3 | Peripheral edema | mild | no | . | no | . |
| control; off OAC | 1 | AF-recurrence | moderate | no | . | . | Start OAC in control arm |
| control; off OAC | 1 | AF-recurrence | moderate | yes | hospitalisation | . | Start OAC in control arm |
| control; off OAC | 1 | AF-recurrence | mild | no | . | . | Start OAC in control arm |
| control; off OAC | 1 | AF-recurrence | moderate | no | . | . | Start OAC in control arm |
|  | 2 | AF-recurrence | moderate | yes | hospitalisation | no | . |
|  | 3 | Perimyocarditis; pericardial effusion | moderate | yes | hospitalisation | no | . |
| control; off OAC | 1 | AF-recurrence | mild | no | . | . | Start OAC in control arm |
|  | 2 | AF-recurrence | mild | no | . | no | . |
|  | 3 | AF-recurrence | moderate | no | . | no | . |
|  | 4 | AF-recurrence | moderate | no | . | no | . |
|  | 5 | Articular gout | moderate | no | . | no | . |
| control; off OAC | 1 | Palpitations | mild | no | . | . | . |
|  | 2 | AF-recurrence | severe | no | . | . | Start OAC in control arm |
|  | 3 | AF-recurrence | severe | no | . | no | . |
|  | 4 | Entropium | severe | yes | intervention | no | . |
|  | 5 | Conjunctivitis | moderate | no | . | no | . |
|  | 6 | AF-recurrence | severe | yes | hospitalisation | no | . |
| cont. control; off OAC | 7 | Pyrosis | mild | no | . | no | . |
|  | 8 | Pyrosis | mild | no | . | no | . |
|  | 9 | Peripheral edema | mild | no | . | no | . |
|  | 10 | Cystitis | moderate | no | . | no | . |
|  | 11 | Dyspnea NYHA functionmal class II | moderate | no | . | no | . |
|  | 12 | Elevated pro-BNP | moderate | no | . | no | . |
| control; off OAC | 1 | Spinal-cord stenosis | mild | no | . | . | . |
|  | 2 | AF-recurrence | moderate | no | . | . | Start OAC in control arm |
|  | 3 | AF-recurrence | mild | yes | hospitalisation | no | . |
|  | 4 | Pleural effusion | moderate | yes | hospitalisation | no | . |
|  | 5 | Pneumonia | moderate | yes | hospitalisation | no | . |
|  | 6 | Pericardial effusion | severe | yes | hospitalisation | no | . |
|  | 7 | Pleural effusion | moderate | yes | hospitalisation | no | . |
|  | 8 | Fall on the head | moderate | no | . | no | . |
| control; off OAC | 1 | AF-recurrence | moderate | yes | hospitalisation | . | Start OAC in control arm |
| control; off OAC | 1 | AF-recurrence | moderate | yes | hospitalisation | . | Start OAC in control arm |
|  | 2 | AF-recurrence | mild | yes | hospitalisation | no | . |
|  | 3 | Worsening of carpal tunnel syndrome | moderate | no | . | no | . |

***Suppl. Table3: Adverse events for group switchers - Intensity, seriousness, relationship. Population: randomized***

AE: adverse event; AF: atrial fibrillation; OAC: oral anticoagulation; SAE: serious adverse event
